# The Temporal Relationship Between Terrestrial Gamma‐Ray Flashes and Associated Optical Pulses From Lightning

**DOI:** 10.1029/2022JD037128

**Published:** 2022-09-01

**Authors:** C. A. Skeie, N. Østgaard, A. Mezentsev, I. Bjørge‐Engeland, M. Marisaldi, N. Lehtinen, V. Reglero, T. Neubert

**Affiliations:** ^1^ Birkeland Centre for Space Science Institute of Physics and Technology University of Bergen Bergen Norway; ^2^ INAF‐OAS Bologna Bologna Italy; ^3^ Imaging Processing Laboratory University of Valencia Valencia Spain; ^4^ National Space Institute Technical University of Denmark Kongens Lyngby Denmark

**Keywords:** terrestrial gamma‐ray flashes, lightning discharge, ASIM, lightning, atmospheric electricity, high‐energy radiation

## Abstract

We present 221 Terrestrial Gamma‐ray Flashes (TGFs) and associated optical pulses observed by the Atmosphere‐Space Interactions Monitor (ASIM) on board the International Space Station. The events were detected between the end of March 2019 and November 2020 and consist of X‐ and gamma‐ray energy detections, as well as photometer data (180–230, 337, and 777 nm) and optical camera data (337 and 777 nm). Using the available ASIM data and applying a consistency check based on TGF characteristics and lightning detections from lightning radio atmospherics close in time, we determine the most likely position of the TGFs in relation to the photometer field of view (FoV), and the association to the observed optical pulses. Out of the 221 events we find 72 events where the TGF and optical data are determined to be associated and inside the photometer FoV. Using the measured TGF durations and the time between the onsets of the TGFs and optical pulses we find: (a) That the TGF onsets are always before or at the same time as the optical pulse onsets (taking into account cloud scattering). (b) A tendency for longer duration TGFs to have longer delays between onsets. (c) Two groups of events: (a) where there is a possible overlap between the TGFs and the optical emissions, as the TGFs last longer than the delay between onsets and (b) where the TGFs and optical emissions do not overlap, as there are long delays between the onsets, which cannot be explained by cloud scattering.

## Key Points

- Terrestrial Gamma‐ray Flashes (TGFs) are produced before or simultaneously with the onset of an optical pulse
- For some events the delay of the optical pulse is too long to be explained by the light scattering in the cloud
- Longer duration TGFs tend to have longer delays between onsets of the TGFs and the optical pulses

## 1 Introduction

Terrestrial Gamma‐ray Flashes (TGFs) are bursts of hard X‐ and gamma‐rays produced via bremsstrahlung from runaway electrons accelerated in the electric fields of thunderstorms (Dwyer, 2012; Gurevich et al., 1992; Moss et al., 2006; Wilson, 1925). The TGFs are reported to typically last a few tens, up to a few hundred microseconds, and have individual photon energies up to ∼40 MeV (Briggs et al., 2013; Fishman et al., 1994; Marisaldi et al., 2010, 2014; Smith et al., 2005). Analysis of the energy spectra of TGFs and lightning radio atmospherics indicate that they are produced below 21 km altitude, most likely between 10 and 15 km (Carlson et al., 2007; Cummer et al., 2014; Dwyer & Smith, 2005; Lindanger et al., 2021; Mailyan et al., 2016; Pu et al., 2019; Stanley et al., 2006; Xu et al., 2012). The underlying mechanism for creating the observed TGF photon fluxes is still unclear. There are two leading models for explaining the observed fluxes based on where and how the electrons are accelerated. Both theories build on electrons being accelerated in electric fields that are strong enough to overcome the friction force of the air, in what is called the runaway process (Wilson, 1925), before being multiplied in a relativistic runaway electron avalanche process (Gurevich et al., 1992). In the first model, an electron flux is created in an avalanche process developing in the large‐scale electric fields within the thunderclouds. Back‐scattered X‐rays created by bremsstrahlung, and positrons created by pair‐production, seed additional electron avalanches in what is called a relativistic feedback mechanism (Dwyer, 2008). In the other model the initial electron flux is created in small, intense, transient overlapping electric fields of streamers, lightning leader and thundercloud electric field. The overlapping electric fields are strong enough to let electrons runaway, and then undergo bremsstrahlung and produce X‐ and gamma‐rays (Celestin & Pasko, 2011; Moss et al., 2006). These two models can also be at play simultaneously, as one does not exclude the other.

TGFs were first reported by Fishman et al. (1994), using the Burst and Transient Source Experiment on the Compton Gamma Ray Observatory. Since then many observations of TGFs have been made, using mostly satellite‐based (Briggs et al., 2010; Marisaldi et al., 2010, 2014; Østgaard, Neubert, et al., 2019; Smith et al., 2005), but also aircraft (Bowers et al., 2018; Smith et al., 2011), and ground based instruments (Abbasi et al., 2018; Dwyer et al., 2004, 2012; Hare et al., 2016; Kereszy et al., 2022; Tran et al., 2015; Wada et al., 2019). The atmosphere‐space interactions monitor (ASIM) is the first instrument specifically designed to observe TGFs, as well as transient luminous events. ASIM is mounted on the Columbus module of the International Space Station (ISS), and has been gathering data since April 2018. ASIM has multiple detectors consisting of high and low energy X‐ and gamma‐ray detectors, photometers, and optical cameras (Neubert et al., 2019).

Past studies using radio data have shown that TGFs likely occur during the early phase of intracloud (IC) lightning (Lu et al., 2010; Pu et al., 2019; Shao et al., 2010; Østgaard et al., 2013, 2021). This is also shown in Lindanger et al. (2022) who used TGF detections paired with optical measurements of lightning activity to show that TGFs are produced during the initial phase of a lightning flash. The sequence of the TGF and optical signal of the flash is still uncertain. Østgaard et al. (2013) were the first to report simultaneous observation of a TGF and optical light from lightning. Using a TGF detected by the Ramaty High Energy Solar Spectroscopic Imager (RHESSI) and optical data from the lightning imaging sensor (LIS) on board the tropical rainfall measuring mission satellite, they conclude that the TGF was produced in the initial stage of an IC lightning propagating upwards in the cloud. Gjesteland et al. (2017) reinvestigated the TGF‐optical sequence using two TGFs detected by RHESSI and optical data from LIS, as well as lightning radio atmospherics from the World Wide Lightning Location Network. However, due to the relative timing uncertainties of the instruments (±1.6 ms), stemming mainly from the time resolution of LIS, they could not determine the sequence of TGF and optical signal of the flash. Alnussirat et al. (2019) also investigated the sequence of TGF and optical signals, using optical data from the Geostationary Lightning Mapper, with a time resolution of 2 ms and TGFs detected by the Fermi Gamma‐ray Burst Monitor. Based on their observations, they find that the TGFs are likely produced during the last stages of the development of the lightning leader channel. However, they could not determine the sequence of TGF‐optical emission itself. More recent observations from ASIM have shown that the majority of TGFs occur before or at the onset of the optical emissions, given the uncertainties in the measurements (Heumesser et al., 2021; Neubert et al., 2020; Østgaard, Neubert, et al., 2019). A relevant aspect in the determination of the sequence of the TGF and optical emissions is the cloud scattering of the optical signals. Satellite detection of optical light from lightning including cloud effects such as scattering, has recently been modeled (Luque et al., 2020; Peterson, 2020). The optical light emitted from lightning in different wavelength bands is associated with different processes in a lightning flash, such as the hot channel of a lightning discharge or streamer activity before the discharge (Chanrion et al., 2019).

In this work we will investigate the temporal relationship between TGFs and optical emissions from lightning. For this purpose we will use a set of upwards directed TGFs with accompanying optical detections observed from space by ASIM. We start by investigating the sequence of TGF and the main optical pulse (defined in Section 3.6), which has been addressed using ASIM before in Østgaard, Neubert, et al. (2019) and Heumesser et al. (2021). The results of Østgaard, Neubert, et al. (2019) were hampered by the relative timing accuracy (±80 µs) between the Modular X‐ and Gamma‐ray Sensor (MXGS) and modular multi‐spectral imaging array (MMIA) instruments. In this paper, we will use a larger data sample from a later period, where the relative accuracy between the instruments has been improved to ±5 µs, and more sophisticated and accurate methodology. Heumesser et al. (2021) also analyzed TGFs and optical data observed by ASIM, and concluded that the sequence of TGF‐optical cannot be addressed due to the uncertainties in timing and the model they used. For this work we have carefully inspected each event and applied a consistency check (outlined in Section 3), where we determine the relationship of each detected TGF and optical pulse. For this purpose we inspected the geolocation source of the radio atmospherics from the lightning discharges, together with the optical detections of lightning and TGF characteristics to determine which of the observed optical pulses are most likely associated with the TGFs. The events where the TGF and optical pulse are found to be associated will then be used to investigate the sequence of the TGF and optical pulse, as well as the relationship between TGF durations and the time delay between TGF and the onset of the optical pulse, to help understanding the processes involved and sequence of events.

## 2 Instruments and Data

The ASIM payload (Neubert et al., 2019) on board the ISS consists of two main instruments: the MMIA and the MXGS. The MMIA (Chanrion et al., 2019) consists of three photometers and two cameras, which are tilted 5° upwards from nadir (toward starboard of ISS) to avoid potential obstructions from payloads on the bottom of the mounting platform. The photometers operate in 180–230 nm (UV), 337 nm (blue) with a 4 nm bandwidth, 777.4 nm (red) with a 5 nm bandwidth, and have a sample rate of 100 kHz. The two optical cameras capture up to 12 frames per second, operate in the 337 and 777 nm bands, and have a 400 × 400 m resolution at nadir. Both the 337 and 777 nm photometers and cameras have a square field of view (FoV) (80° diagonal), while the UV photometer has a circular FoV (80° diameter). As we are only using the 777 and 337 nm band in this study we will refer to the square FoV as MMIA FoV throughout this paper. To prevent damage by sunlight, the MMIA instrument is only active during night time. The MXGS (Østgaard, Balling, et al., 2019) consists of a high and a low energy detector (LED). The high‐energy detector (HED) is always active while outside the South Atlantic Anomaly, detects energies between 300 keV and 30 MeV, and has a time resolution of 28.7 ns. The LED is only active during night, due to optical photon contamination during day time, detects energies between ∼50 and 400 keV, and has a resolution of 1 µs. The ASIM instrument includes a cross triggering system between MXGS and MMIA, such that if either instrument triggers data from both will be kept for a period of ∼2 s, centered on the trigger time. The relative timing accuracy between the MXGS and MMIA instrument was ±80 µs until a software update in April 2019, which reduced it to its nominal performance of ±5 µs. Due to a non‐optimal timing interface between the ISS and the ASIM instrument, the absolute timing accuracy is found to be ∼−10 to +40 ms (determined using lightning detection location from lightning radio atmospherics). This timing accuracy can be improved for some events by using lightning detections together with optical data to reduce the absolute timing accuracy to ±1 ms.

Lightning radio atmospherics used in this work is provided by Vaisala's Global Lightning Detection Network (GLD360) and give us time and location data of lightning flashes. GLD360 is a ground‐based very‐low‐frequency (VLF) and lower part of low‐frequency (LF) radio lightning detection network which employs time of arrival and magnetic direction finding at each sensor to determine the location of individual lightning discharges. The expected GLD360 detection efficiency is ∼75%–85% for cloud‐to‐ground flashes, ∼40%–50% for IC pulses, with ∼2–6 km uncertainty in median location accuracy (Demetriades et al., 2010; Said & Murphy, 2016).

## 3 Methodology

Between end of March 2019 and November 2020 we have observed 221 TGFs, where also optical data from MMIA are available with a relative timing accuracy of ±5 µs. To determine the association between the TGFs and the optical pulses we investigated the photometer data in three main steps. (a) We search for an optical pulse in the 337 and 777 nm optical band within 5 ms of the TGF. If there is no optical pulse the TGF is most likely outside the MMIA FoV and we exclude the event. (b) For the remaining events the MMIA FoV is determined and the surrounding lightning activity within 15 min of the TGF is investigated to determine a possible location of the TGF. (c) A consistency check is performed (as outlined in Section 3.3) using the surrounding lightning activity, camera images (83.3 ms resolution), and TGF characteristics, such as number of counts and their energies.

### 3.1 MMIA Field of View

**Figure 1:**
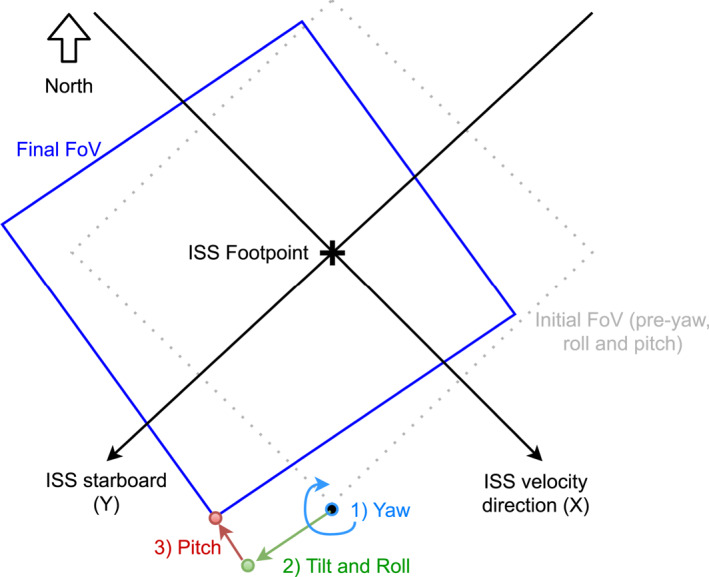
Illustration of how to determine the corner‐point of the modular multi‐spectral imaging array (MMIA) field of view (FoV) projected from the International Space Station (ISS) to ground level on Earth in three steps. 1) Rotation due to ISS yaw angle (±*Z*‐direction), 2) move according to roll angle and the 5° tilt of the MMIA instrument in the roll direction (±*Y*‐direction). 3) Move according to the pitch angle (±*X*‐direction).

To determine the square MMIA photometer FoV we first interpolate the ISS foot point at the time of the TGF, using the closest ISS locations before and after the TGF, as well as the ISS velocity and the difference in time between the TGF detection and the two points. From the ISS foot‐point we map out the 337 and 777 nm photometer FoV, which is a square with 80° diagonal for both photometers (Chanrion et al., 2019), with the sides going along and across the ISS direction of travel (*X* and *Y* in Figure 1). This is done by using eight points, namely the four corners of the square and points at the middle of each side. These eight points are then shifted using yaw, roll, and pitch angles of the ISS at the time of the TGF, as well as the 5° tilt in the roll direction, as illustrated in Figure 1. The photometer FoV was compared with coastline camera images, acquired specifically to optimize the pointing accuracy. The difference in the determined FoV and coastal camera images was found to be at most ∼20 km, which is acceptable for the purposes of this study.

### 3.2 Lightning Detections

Using the location data from lightning radio atmospherics (detected by GLD360) in the surrounding area from within ±1,000 s we get three different scenarios. (a) All lightning activity is outside the MMIA FoV, meaning the TGF is most likely from outside the FoV. (b) All lightning activity is inside the MMIA FoV, meaning the TGF is most likely from inside the FoV. (c) There is lightning activity both inside and outside the MMIA FoV. For all of these groups of events we apply the consistency check (see Section 3.3), where the lightning activity is used together with (a) TGFs individual photon energies and fluence and (b) camera images.

The GLD360 detections are also used to improve the absolute timing accuracy of ASIM, which is found to be −10 to +40 ms before improvement. For many of the events we can improve the absolute timing of ASIM by time alignment of the measured optical pulses and GLD360 detections. This is done by first finding the ISS time of the GLD360 detections, by adding the lights' travel time from source to the ISS, to the given GLD360 detection time. We then use multiple triggers of MMIA data (up to three, consisting of the trigger containing the TGF and one trigger before and after the trigger containing the TGF) to align as many optical pulse peaks as possible to the GLD360 detections. A minimum of two alignments is required, with a minimum of one optical pulse aligned from the MMIA trigger containing the TGF.

Aligning the GLD360 detections to the optical pulse peaks is practical, although this approach does not take into account the time delay due to light scattering through the clouds. However, given the typical rise times of the optical pulses, this is well within the error of the method. The lightning detection locations and camera images are then checked for consistency. Using this technique we can get the absolute timing accuracy between MMIA and the GLD360 detections down to ±1 ms. This method of improving absolute timing accuracy has already been implemented in Maiorana et al. (2021), Lindanger et al. (2022), and independently developed and applied in Heumesser et al. (2021). Using the method outlined here we found 95 alignments for the total sample of events.

### 3.3 Consistency Check

A consistency check is performed to determine if the TGFs are likely to be within the MMIA FoV and have an association with the optical pulses. For this purpose we use the TGF fluence and individual photon energies, and compare to optical camera and lightning activity. For the consistency check we consider a TGF emission half‐cone of 30–40° without tilt. The TGF fluence is expected to be reduced as the distance between the TGF and ISS‐footpoint increases. This is due to the scattering of photons in the atmosphere and the increasing distance (1/*R* ^2^ effect). Furthermore, we expect TGFs observed within the production cone to have more high energy (above energy channel 1,000, which is approximately 10 MeV) counts than the TGFs from outside the production cone. For TGFs observed outside the initial production cone the photons will have undergone Compton scattering and have reduced energies (Carlson et al., 2007; Gjesteland et al., 2011; Lindanger et al., 2021). This means the TGFs with no or very few counts with high energies are more likely to be produced outside the MMIA FoV, because the half‐cone angle (30–40°) is similar to the MMIA FoV (diagonal angle of 40°). We also investigate the lightning activity surrounding the TGF and check for a GLD360 detection associated to the TGF and optical pulse. If such a pulse is found we compare the location of the GLD360 detection to the optical camera, as well as the TGF characteristics. We do not use any of these assumptions as hard limitations, but as an indicator to differentiate TGFs inside the MMIA FoV from those outside the MMIA FoV.

### 3.4 Event Examples

Following are examples that show events where the consistency check is used to help determine the TGF‐optical pulse association.

**Figure 2:**
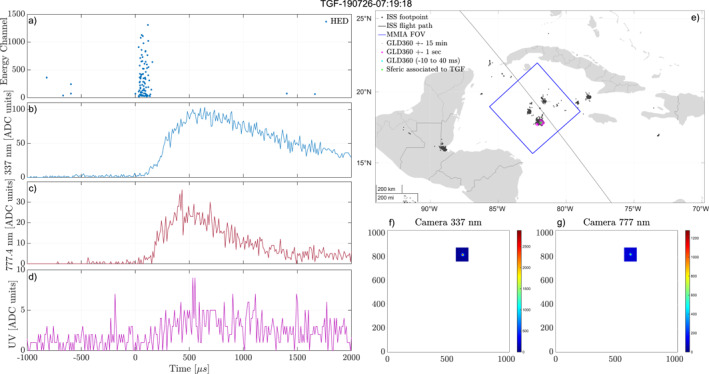
Example of an event where we have determined the Terrestrial Gamma‐ray Flash (TGF) to be inside the modular multi‐spectral imaging array (MMIA) field of view (FoV) and associated to the observed optical pulse. (a) Detected counts in high‐energy detector (HED). (b–d) Light curves from the photometers, 337, 777, and 180–230 nm. (e) Map of the area around the International Space Station (ISS) footpoint. The blue square shows the MMIA FoV, the black dots are lightning activity within ±15 min of the TGF, the magenta stars are lightning activity within a second of the TGF, and the green star shows the lightning detection aligned to the optical pulse. (f, g) Camera 337 and 777 nm show the optical camera images from the frame containing the TGF (*Y*‐axis is along the flight path of the ISS, which is always eastwards/right in the images).

Figure 2 shows an example of an event where we have determined that the TGF and optical pulse is associated. For this event enough GLD360 detections could be aligned with optical pulses so that the total timing accuracy was reduced to ±1 ms. As seen panel (e) of the figure, most lightning activity is inside the MMIA FoV, with multiple lightning detections within 1 s of the TGF clustered at the same area. The magenta stars show the GLD360 detections within 1 s of the TGF time, with the green star showing the GLD360 detection found to be aligned in time to the optical pulse. No other GLD360 detections were found to be within the −10 to +40 ms window (blue stars), corresponding to the total timing accuracy of ASIM. The camera images (f and g) support that the optical pulses come from the same location as the GLD360 detections. Panel (a) shows the counts, with energies from the HED. The TGF has a hard spectrum (five counts above channel 1,000) with many counts (97) in a short time interval (∼100 µs), which is consistent with a TGF within a small radial distance ∼200–300 km to the ISS foot‐point, that is, within the MMIA FoV. ISS is moving eastward and the camera images have *Y*‐axis along the path and *X*‐axis toward starboard. This all indicates that the TGF is from within the MMIA FoV and associated to the observed optical pulse.

**Figure 3:**
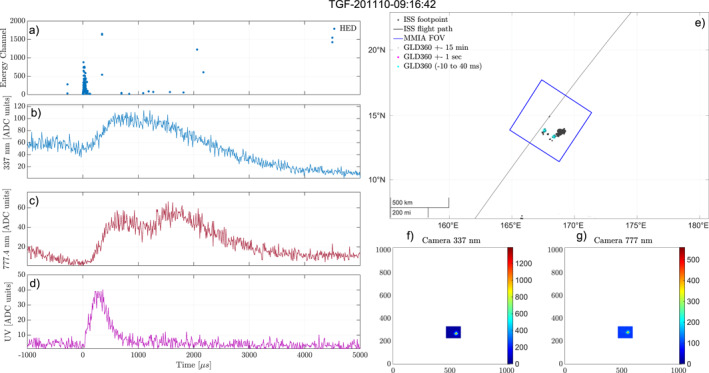
Example of an event where we have determined the Terrestrial Gamma‐ray Flash (TGF) to be inside the Modular multi‐spectral imaging array (MMIA) field of view (FoV) and associated to the observed optical pulse, and there is only lightning activity from within the FoV. (a) Detected counts in high‐energy detector (HED). (b–d) Light curves from the photometers, 337, 777, and 180–230 nm. (e) Map of the area around the International Space Station (ISS) footpoint. The blue square shows the MMIA FoV, the black dots are lightning activity within ±15 min of the TGF, the magenta stars are lightning activity within a second of the TGF, and the green star shows the lightning detection aligned to the optical pulse. (f, g) Cameras 337 and 777 nm show the optical camera images from the frame containing the TGF (*Y*‐axis is along the flight path of the ISS, which is always eastwards/right in the images).

Figure 3 shows another example of an event where the TGF is determined to be within the MMIA FoV. For this event there is only lightning activity within the MMIA FoV. There were not enough GLD360 detections to improve the timing accuracy for this event. Two GLD360 detections are seen inside the MMIA FoV within the minimum and maximum of the absolute timing correction (blue stars). One of the detections is most likely an ionospheric reflection, considering the time, distance, and peak currents (opposite but similar magnitude). This is also supported by the camera images, where only one active area is seen. An ELVE (Emission of Light and Very Low Frequency perturbations due to Electromagnetic Pulse Sources) event is observed (UV pulse starting at 0 µs) at the same time as the TGF. Thirteen such ELVEs were found in this data set, these events are also a part of the data set analyzed and presented in Bjørge‐Engeland et al. (2022).

A total of 72 events was found, where we could determine an association between the TGF and the optical pulse such as the events shown in Figures 2 and 3. Fourty five absolute timing corrections were determined for these 72 events, where 33 ended up with a GLD360 detection aligned with the optical pulse associated to the TGFs.

**Figure 4:**
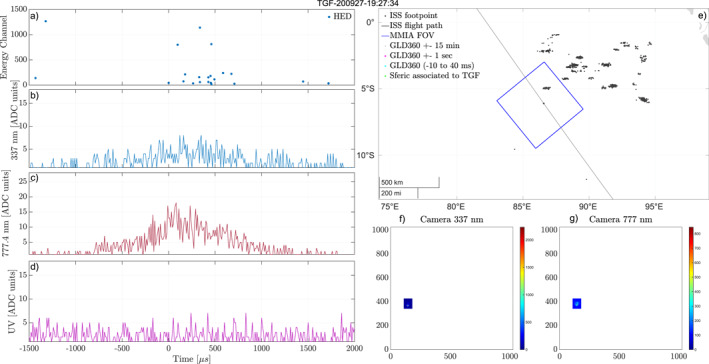
Example of an event where we have determined the Terrestrial Gamma‐ray Flash (TGF) to be outside the modular multi‐spectral imaging array (MMIA) field of view (FoV) and is therefore not associated to the observed optical pulse. (a) Detected counts in high‐energy detector (HED). (b–d) Light curves from the photometers, 337, 777, and 180–230 nm. (e) Map of the area around the International Space Station (ISS) footpoint. The blue square shows the MMIA FoV, the black dots are lightning activity within ±15 min of the TGF. No lightning detections are within 1 min of the detected TGF (magenta, blue, or green stars). (f, g) Camera 337 and 777 nm show the optical camera images from the frame containing the TGF (up is along the flight path of the ISS, which is always eastwards/right in the images).

Figure 4 shows an event for which we have determined that the TGF is not from within the MMIA FoV, and therefore the observed optical pulse is not associated with the TGF. The lightning activity map (e) shows there are many centers of activity outside the MMIA FoV, but no lightning activity within 1 s of the TGF (magenta, blue, or green stars). The camera images (f–g) show that the optical pulses come from a location close to the ISS foot‐point. The TGF (a) is found to be long, with relatively few counts in HED (19) and only one count above energy channel 1,000. The TGF characteristics in this instance do not match our expectations of a TGF found inside the MMIA FoV and close to the ISS foot‐point. In this case it is likely that the TGF is produced in one of the active areas outside the FoV and is not associated with the optical pulses we observe. We found 57 events of this type, where the TGF is most likely outside the FoV, and consequently not included in our study.

**Figure 5:**
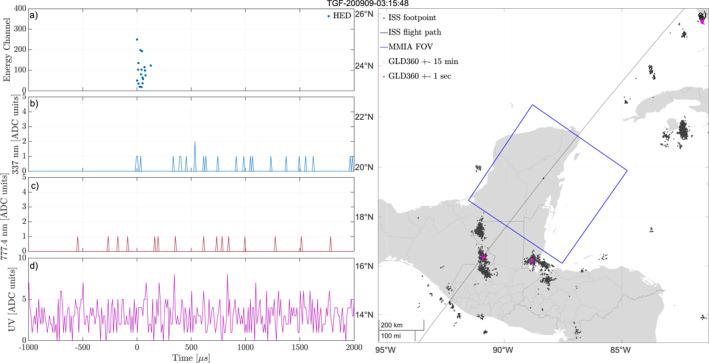
Example of an event where the Terrestrial Gamma‐ray Flash (TGF) is outside modular multi‐spectral imaging array (MMIA) field of view (FoV), and there is no optical pulse observed in the photometers. (a) Detected counts in high‐energy detector (HED). (b–d) Light curves from the photometers, 337, 777, and 180–230 nm. (e) Map of the area around the International Space Station (ISS) footpoint. The blue square shows the MMIA FoV, the black dots are lightning activity within ±15 min of the TGF, the magenta stars show lightning activity within a second of the TGF.

The last group of events is the 88 events excluded in the beginning where we have no association, as there is no observed optical pulse within ±5 ms of the TGF. Figure 5 shows one of these events, with no lightning activity within the MMIA FoV as further support. Some lightning activity is observed within 1 s of the TGF just outside the MMIA FoV, shown as the three magenta stars. The TGF has few counts, with energies below channel 300, which is consistent with being produced outside the MMIA FoV.

For four events there are difficulties in determining optical pulse onset or TGF association to the optical pulse. This is due to there either being multiple TGFs within the 5 ms time window, but only one optical pulse, or difficulties identifying the pre‐activity and determining the onset of the optical pulse. As we do not want to make an assumption on which TGF is associated to the optical pulse, or what is pre‐activity and main optical pulse, we decided to exclude these events going forward. Table 1 shows a summary of the number of events in each category.

**Table 1:** Summary of Events

Inside FoV (# time alignments)	Most likely outside FoV	No optical pulse	Non‐determinable
72 (45)	57	88	4

*Note*. FoV, field of view.

### 3.5 TGF Durations

The TGF durations were determined using an algorithm to find a “core duration” defined as the shortest time interval which includes 90% of the counts (*t*_core90_). The algorithm works by first binning the counts around TGF time into 40 µs bins and selecting the bin with the most counts as a starting point. Thereafter we go backwards and include any count that is within 100 µs of the previously included count. The first photon of the TGF is then found where there is more than 100 µs between the current count and the one before it. This same approach is also applied from the starting position and forwards in time, to find the end of the TGF. A sliding window is then used to find the shortest duration that contains 90% of the counts (rounded up) between the start and end counts. As this method runs the risk of including cosmic showers and solar protons we perform a manual inspection of the events for the purpose of removing these counts. To determine the uncertainties in TGF *t*_core90_ durations we chose to add the closest count in time before and after the determined TGF counts in the method outlined above (these counts will be more than 100 µs before/after the first/last count). We then determine the *t*_core90_ duration of this sample and use the difference in duration compared to the original *t*_core90_ as the uncertainty.

### 3.6 Optical Pulse Onsets

To determine the onset of the optical pulse we determined several separate linear fits to different parts of the optical pulse. We used the intersects of linear fits of the sharpest rise in the optical pulse and the activity before this rise. To make these fits we mainly used the 777 nm light curves, but for some events a good fit could not be made to the 777 nm light curves, so the 337 nm light curves were used instead. The red light (777 nm) is preferred as it is thought to be mainly associated to atomic oxygen (OI) which may only exist (after dissociation of O_2_) in the heated lightning leader channel (Chanrion et al., 2019), whereas the blue light (337 nm) is associated to the streamer activity, namely N_2_ second positive band group radiation caused by excitation by supra‐thermal electrons. The 337 band might also detect some UV emissions, such as from ELVEs.

**Figure 6:**
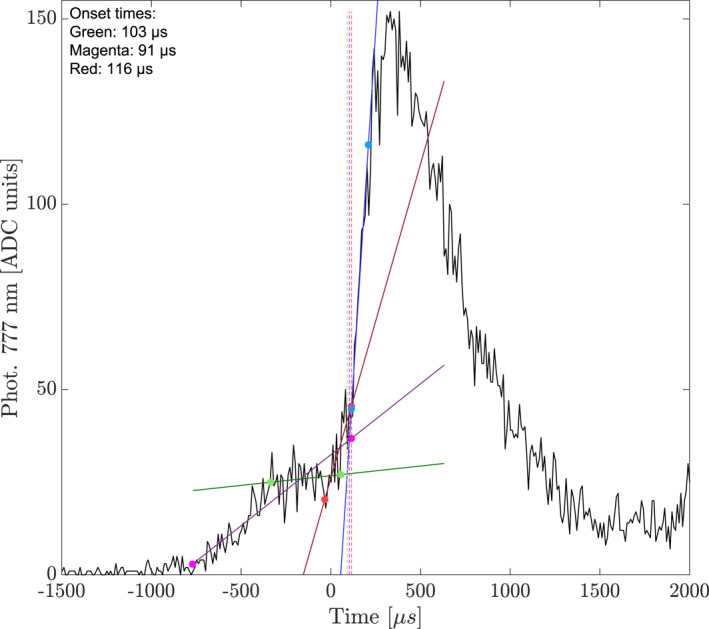
Example of optical pulse onset determination. The solid colored lines show the different linear fits: green, purple, and red lines show fits for different parts of the pre‐activity data, while the blue line shows a fit to the sharpest increase of the optical pulse. The dots on the fit lines represent the start and end of the data used in the fitting. The dotted lines represent the intersects between the pre‐activity fits (green, purple, and red) and the fit for the sharpest increase.

For each event we separate the optical pulse activity into pre‐activity and main rise (sharpest increase). One linear fit was made for the sharpest rise of the optical pulse, while we use three fits for the pre‐activity. The pre‐activity is thought to be optical emissions from the propagating lightning leader (Østgaard, Neubert, et al., 2019) and can be seen in Figure 6 as the relatively slow increase after −1,000 µs and until ∼+100 µs. For some cases we found it necessary to make two fits to the sharpest increase part, as the pulse was irregular with a separate increase between the sharpest increase and the pre‐activity. The average of the intersects between the main rise fit lines and the multiple pre‐activity fit lines is used as the optical onset time, with the difference between the minimum and maximum times of these intersects used to define the uncertainties. The uncertainties range from 1 µs to ∼100 µs, with an average of 13 µs. Figure 6 shows an event where we have determined four linear fits. The solid colored lines show the different linear fits: blue for the sharpest increase in the optical pulse, magenta, red, and green for different segments of pre‐activity. The colored dots show the start and end of the data used in the fit. The dotted lines show the intersects from the fits of the pre‐activity to that of the sharpest increase. As seen in the figure, the pre‐activity has a linear trend, but can be broken into three parts for this particular event. The magenta line shows the total pre‐activity, while the green line uses the data points at what appears to be a plateau in the pre‐activity. Lastly, the red line used the data points in the last part of the pre‐activity where we have a steeper increase than for the previous parts of the pre‐activity.

## 4 Results and Discussion

During the period between end of March 2019 and November 2020, 221 events were detected with both MMIA and MXGS data. From these events 88 had no optical pulse within ±5 ms of the TGFs. Using the method outlined in Section 3 we determined that for 72 of the events the TGFs are inside the MMIA FoV, and associated to the observed optical signal. Using the lightning radio atmospherics from GLD360 we find that the absolute timing can be improved for 95 of the 221 events, which includes 45 timing corrections for the events where TGFs are determined to most likely be inside the MMIA FoV. The mean timing correction was found to be ∼+14 ms, varying from −11.5 to +40 ms. Going forward, all results refer to the sample of 72 events determined to most likely be within the MMIA FoV.

**Figure 7:**
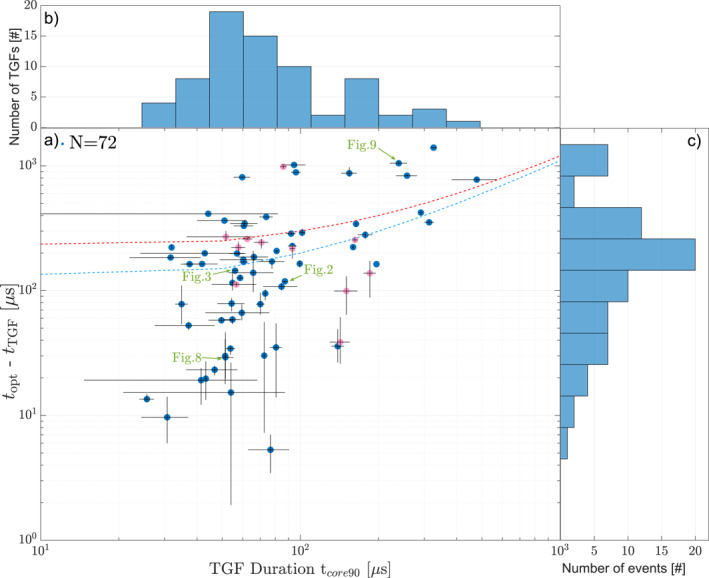
(a) Plot showing the difference in Terrestrial Gamma‐ray Flash (TGF) and optical onset times compared to TGF durations, with accompanying uncertainties. The blue dots show the events with onsets determined from the 777 nm optical pulse and the pink dots show the events where onsets were determined from the 337 nm optical pulse. Green arrows refer to events that are shown in separate Figures. The dotted red line shows the maximum expected optical delay due to cloud scattering, of 200 µs, and the dotted blue line shows the expected value (∼100 µs). (b) TGF *t*_core90_ durations in 10 logarithmic bins. (c) Histogram of 10 logarithmic bins showing determined delays between TGF and optical signal.

Using the TGF *t*_core90_ durations and the delay between onsets we plot the relationship between the two in panel (a) of Figure 7. The blue and pink dots show this relationship, where blue dots have the optical onset determined from 777 nm, and the pink dots have optical onsets determined from 337 nm. The TGFs showing large uncertainties in duration have typically low number of counts, for which our method of determining the TGF duration is less robust. The expected delay of the observed optical onsets due to scatting in the cloud was modeled by Luque et al. (2020). Using a uniform cloud with typical value for particle radius of 10 μm and particle density of 100 cm^−3^ the delay of the 777 pulse would be 100 µs for a 6 km thick cloud and 200 µs for 8.5 km thick cloud. Considering a cloud top of 15 km, a delay of up to 100 µs seems realistic, with expectations that TGFs from these altitudes (above 9 km) are easily observable. A 200 µs delay due to a 8.5 km thick cloud would place the source very deep in the cloud (∼6.5 km), making it more difficult to observe all but the most powerful TGFs. 200 µs is therefore used as an upper limit of delay due to scattering. Figure 7a shows the lines corresponding to these two cloud scattering delays added to the TGF durations (dotted red and blue lines). The plot shows a tendency for longer‐duration TGFs to have longer optical delays.

Panel (c) of the figure shows the distribution of the optical onset delays with respect to the TGF *t*_core90_ start time. The delays are calculated as the duration from the first photon of the TGF *t*_core90_ duration and the onset of the main optical pulse. The data is binned to 10 logarithmic bins. The median determined delay between onsets is 190 µs, with only nine events having delays over ∼500 µs. None of the events are observed to have a negative delay, that is, there are no observed events where the optical pulse onset precedes the onset of the TGF. This has been ambiguous in previous work with ASIM data, but has been improved here due to the new and larger sample of events with better relative timing accuracy and our method of determining that the location of TGFs is inside the MMIA FoV and association to optical pulses. The sequence of events presented here support the sequence presented in Østgaard, Neubert, et al. (2019). Our determined delay between onsets is a bit different to what has previously been reported using ASIM data. Heumesser et al. (2021) use ASIM data from between June 2018 and October 2019 to determine characteristics of the optical pulses associated to TGFs. Heumesser et al. (2021) shows in Figure 3, panel (a), events with negative delays, with a mean source time of −10 µs and outliers with more than 4 ms delays. We have shown in this work, using the methods outlined that there are no negative values between onsets, albeit with a different methodology and somewhat different data set (we only use ASIM data from after end of March 2019, when the MXGS‐MMIA uncertainty was reduced to ±5 µs). However, like Heumesser et al. (2021) we see the same trend with the majority of events having less than ∼2–300 µs delays between onsets and a similar group of outliers at around ∼800–1,200 µs.

Panel (b) in Figure 7 shows the TGF *t*_core90_ durations of the 72 events within MMIA FoV, binned to 10 logarithmic bins. The average *t*_core90_ duration for the 72 TGFs determined to be within MMIA FoV is found to be ∼100 µs, with a median of 66 µs, which is similar to previously reported ASIM detected TGFs by Østgaard, Neubert, et al. (2019) and Bjørge‐Engeland et al. (2022).

All events are found to have the optical signal after the TGF onset, with the events below the dotted blue line (100 µs scattering delay) being consistent with optical emissions produced simultaneously with the TGF (taking into account typical delay due to cloud scattering and uncertainties). There are 37 events (∼51%) which are below the 100 µs delay line (dotted blue), meaning the TGF and the source of the optical pulse could be simultaneous. Fifteen events (∼21%) are above the 200 µs delay line, where the delay of the observed optical pulses cannot be explained by scattering from the cloud alone. Twenty events (∼28%) are between the two delay lines, which means that we cannot rule out that the TGFs and sources of the optical pulses are simultaneous. However, we think it is unlikely that so many powerful TGFs are detected from so deep in the cloud. The next subsections will discuss the events that are below the 100 µs delay line (dotted blue) and the events above the 200 µs delay line (dotted red).

### 4.1 Events Where Optical Onset Delay Is Less Than 100 µs

**Figure 8:**
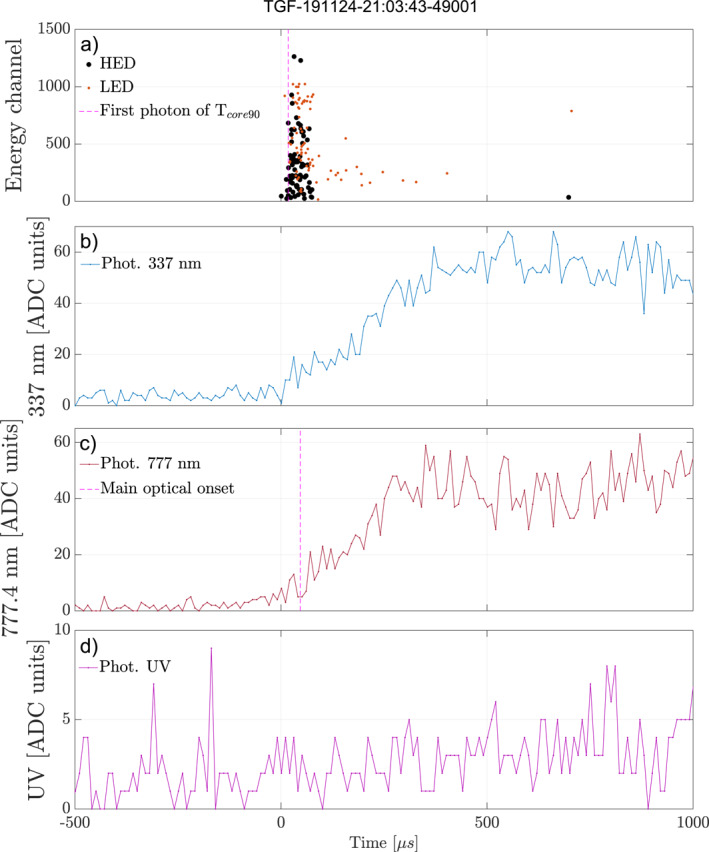
Plot showing the detection of a Terrestrial Gamma‐ray Flash (TGF) that continues after the onset of the optical pulse. (a) Shows the high‐energy detector (HED) and low energy detector (LED) counts given in energy channels. The dotted magenta line shows the first photon of the TGF *t*_core90_ duration. Panels (b–d) show the photometer data of 337, 777, and 180–230 nm respectively. The dotted magenta line in the panel (c) shows the determined onset of the optical pulse.

In Figure 7a there are 37 events (∼51%) where the delay is less than 100 µs, meaning that the TGF and the source of the optical pulse could be simultaneous, when a more realistic delay due to scattering in the cloud is taken into account. For many short TGFs it is difficult to firmly establish whether the TGFs end before the onset of the optical pulse or not, due to the uncertainties of the method. For some events with longer duration the TGFs appear to be continuing after the onset of the optical pulse, suggesting that the conditions for producing runaway electrons as well as X‐ and gamma‐rays (electric field, voltage drop, and seed particles) could still exist after the current through the leader channel occurs. However, most of these events have very weak optical signatures, and we can therefore not make a strong statement based on these outliers. Figure 8 shows one of these events (marked in Figure 7 with a green arrow). Panel (a) shows the HED and LED detections, with the dotted magenta line showing the first photon of the TGF *t*_core90_ duration. The three lower panels (b–d) show the photometer data from 337, 777, and 180–230 nm. The dotted magenta line in the 777 nm panel shows the average onset determined from the method described in Section 3.6. As seen in Figure 8 the TGF is not over at the time of the optical pulse onset. The HED counts (black dots) found after the optical pulse onsets are also high energy, so that they cannot be said to be Compton scattered photons (which can be seen in LED, after the end of the black dots). This means that the TGF is still ongoing at the onset of the optical pulse, meaning the large electric field needed for TGF production is still in place when the current surge responsible for the optical pulse starts.

### 4.2 Events Where Optical Onset Delay Is by More Than 200 µs

**Figure 9:**
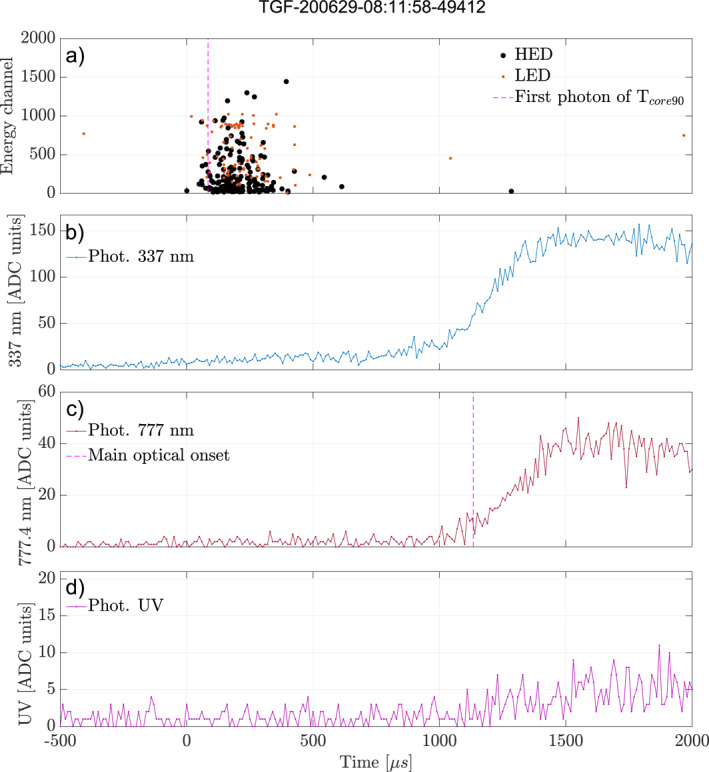
Plot showing the detection of a Terrestrial Gamma‐ray Flash (TGF) that has an unusual long delay between onset of TGF and optical pulse. (a) Shows the high‐energy detector (HED) and low energy detector (LED) counts given in energy channels. The dotted magenta line shows the first photon of the TGF *t*_core90_ duration. Panels (b–d) show the photometer data of 337, 777, and 180–230 nm, respectively. The dotted magenta line in panel (c) shows the determined onset of the optical pulse.

In Figure 7 there are 15 (∼21%) events that are above the 200 µs delay line (dotted red). This means that the conditions for TGF production are no longer present and a time interval (few hundreds up to a thousand microseconds) before the leader current occur is observed. Figure 9 shows one of these 15 events, where panel (a) shows the HED and LED counts of the TGF and the three lower panels (b–d) show the photometer data for 337, 777, and 180–230 nm. The dotted magenta lines show the onset of the TGF *t*_core90_ (a) and optical pulse (c) and ∼1 ms delay between the two can be seen. There is ∼600 µs from the end of the TGF to the onset of the optical pulse.

The connection between TGF and the leader current is a complex problem. This study shows that TGFs start before or simultaneous with the optical onset source. The study also shows that there is a group of events where the TGFs end (∼21%) before the leader current occurs.

## 5 Summary of Results

Based on the 72 events where we have optical pulses associated with TGFs, we find the following:

1. All events have TGF onsets before the onset of the optical pulses
2. 37 (∼51%) of the events are below the 100 µs delay line and therefore have TGF onsets before or simultaneous with the onset of the optical pulse, taking into account the light scattering in the cloud
  - Some of these events could have TGFs ending after the onset of the optical pulse
3. 15 (∼21%) of the events (above the 200 µs delay line) have longer delays than can be explained by cloud scattering, which means that the TGFs end before the leader current occurs
4. 20 (∼28%) of the events are between the two delay lines, these events are compatible with TGF onsets before or simultaneous with the onset of the optical pulse, taking into account a 200 µs delay due to light scattering in the cloud
5. Longer duration TGFs tend to have longer delays between the onset of TGF and the optical pulse

## Data Availability

ASIM data used in this study are publicly available from the ASIM Science Data Center (https://asdc.space.dtu.dk). Values determined in this paper and presentations of the 221 events are available at Zenodo: https://doi.org/10.5281/zenodo.6992464 (Skeie, 2022).
